# Early Contrast Enhancement: A novel magnetic resonance imaging biomarker of pleural malignancy

**DOI:** 10.1016/j.lungcan.2018.01.014

**Published:** 2018-04

**Authors:** Selina Tsim, Catherine A. Humphreys, Gordon W. Cowell, David B. Stobo, Colin Noble, Rosemary Woodward, Caroline A. Kelly, Laura Alexander, John E. Foster, Craig Dick, Kevin G. Blyth

**Affiliations:** a Pleural Disease Unit, Queen Elizabeth University Hospital, Glasgow, UK; b Institute of Cancer Sciences, University of Glasgow, UK; c Department of Pathology, Queen Elizabeth University Hospital, Glasgow, UK; d Department of Radiology, Queen Elizabeth University Hospital, Glasgow, UK; e Department of Radiology, Glasgow Royal Infirmary, Glasgow, UK; f Clinical Research Imaging Facility, Queen Elizabeth University Hospital, Glasgow, UK; g Cancer Research UK Clinical Trials Unit, Institute of Cancer Sciences, University of Glasgow, Glasgow, UK; h Institute of Infection, Immunity & Inflammmation, University of Glasgow, UK

**Keywords:** ECE, Early Contrast Enhancement, ROISIG, Region of Interest Signal Intensity Gradient, MSIG, Mean Signal Intensity Gradient, Mesothelioma, Pleural malignancy, Pleural effusion, Magnetic resonance imaging, Biomarker

## Abstract

**Introduction:**

Pleural Malignancy (PM) is often occult on subjective radiological assessment. We sought to define a novel, semi-objective Magnetic Resonance Imaging (MRI) biomarker of PM, targeted to increased tumour microvessel density (MVD) and applicable to minimal pleural thickening.

**Materials and methods:**

60 consecutive patients with suspected PM underwent contrast-enhanced 3-T MRI then pleural biopsy. In 58/60, parietal pleura signal intensity (SI) was measured in multiple regions of interest (ROI) at multiple time-points, generating ROI SI/time curves and Mean SI gradient (MSIG: SI increment/time). The diagnostic performance of Early Contrast Enhancement (ECE; which was defined as a SI peak in at least one ROI at or before 4.5 min) was compared with subjective MRI and Computed Tomography (CT) morphology results. MSIG was correlated against tumour MVD (based on Factor VIII immunostain) in 31 patients with Mesothelioma.

**Results:**

71% (41/58) patients had PM. Pleural thickening was <10 mm in 49/58 (84%). ECE sensitivity was 83% (95% CI 61–94%), specificity 83% (95% CI 68–91%), positive predictive value 68% (95% CI 47–84%), negative predictive value 92% (78–97%). ECE performance was similar or superior to subjective CT and MRI. MSIG correlated with MVD (r = 0.4258, p = .02).

**Discussion:**

ECE is a semi-objective, perfusion-based biomarker of PM, measurable in minimal pleural thickening. Further studies are warranted.

## Highlights

• A novel MRI biomarker of pleural malignancy is described – Early Contrast Enhancement.
• Early Contrast Enhancement is a semi-objective, perfusion-based biomarker.
• Early Contrast Enhancement can be measured in patients with minimal pleural thickening.

## 1 Introduction

Radiological detection of pleural malignancy (PM) is frequently difficult because overt pleural tumour may be occult and pleural effusion may be the dominant, or only, feature [1]. This is particularly true in early stage Malignant Pleural Mesothelioma (MPM), where extra-pleural malignant features are frequently absent. Recent studies reflect these challenges, reporting low sensitivity and considerable inter-observer variation, using Computed Tomography (CT) in a routine clinical setting [2,3].

Dynamic contrast-enhanced CT (DCE-CT) or perfusion CT allows assessment of tumour microcirculation, including blood flow and capillary permeability [4]. Previous studies have demonstrated potential utility in assessment of pulmonary nodules and MPM [[5], [6], [7]]. A major advantage of perfusion CT is its widespread availability, however, the multiplicity of protocols using different mathematical models [8] and high radiation burden have limited its widespread use in routine clinical practice to date [9,10].

Positron Emission Tomography (PET)-CT is a useful modality in staging of thoracic malignancy, particularly when evaluating nodal and distant metastatic disease, however its role in the differentiation of benign and malignant pleural effusions is less well-established. Recent meta-analyses have demonstrated a pooled sensitivity of 81% and specificity 74% for detecting PM, with considerable variation between studies [11]. Tumours with low metabolic activity, such as early stage epithelioid MPM are more likely to have a false negative PET-CT and false positives in patients with inflammatory pleuritis, TB pleuritis and previous pleurodesis are well recognised [[12], [13], [14]].

Efficient diagnosis is further complicated by the variable performance of pleural cytology, which has a mean sensitivity of 60% (depending on tumour type) but extremely low negative predictive value (NPV) in MPM, for which histological confirmation remains mandatory in most centres. Thoracoscopy offers excellent sensitivity, but is associated with additional healthcare costs, increased procedure-related risk and limited availability. Diagnostic delays are therefore common in patients with PM.

A reliable, non-invasive imaging marker would be a major clinical advance, allowing pathway rationalization and early direction of patients to thoracoscopy, including those with probable early-stage MPM. Ideally, this should be objective, to improve reporting consistency, and applicable to patients with minimal pleural thickening, since it would offer limited additional value in cases with overt nodular disease. Magnetic Resonance Imaging (MRI) is an attractive modality for this purpose, facilitating both high contrast-resolution anatomical and perfusion studies. Dynamic-Contrast-Enhanced MRI (DCE-MRI) exploits the pathognomonic increase in blood vessel density typical of cancers and related to neoangiogenesis [[15], [16], [17], [18]]. DCE-MRI has previously been used to reliably differentiate malignant from benign breast and prostate lesions [18,19] and to generate prognostic [[15], [16], [17], [18]] and predictive (regarding response to anti-angiogenic chemotherapy) [20] data in advanced MPM. However, DCE-MRI requires bulky pleural tumour for application, making it unsuitable for early diagnostics. Neoangiogenesis is an early biological event in tumourigenesis [21] and is therefore likely to be present in patients with smaller pleural tumour volumes. We hypothesised that novel MRI methodology targeted to increased micro-vessel density (MVD) could accurately identify patients with PM, including those with minimal pleural thickening.

## 2 Materials and methods

### 2.1 Study design

A prospective cohort study was performed, incorporating patients recruited to an MRI pilot study and the DIAPHRAGM study (ISRCTN registration 10079972), which contained an MRI sub-study. [22] Table 1 summarises the objectives and *a priori* outcome measures. Consecutive patients presenting to the Glasgow Pleural Disease Unit were invited to participate (January 2013 − October 2016), based on the following eligibility criteria.

• Inclusion criteria: 1) suspected PM requiring histological sampling (by thoracoscopy or image-guided pleural biopsy); this was defined by the presence of a unilateral pleural effusion, pleural thickening or pleural mass lesion and non-diagnostic pleural fluid analysis (including negative fluid cytology); 2) sufficient fitness for pleural biopsy 3) informed written consent.
• Exclusion criteria: 1) pregnancy; 2) gadolinium allergy; 3) renal impairment (eGFR <30 ml/min); 4) known MRI contraindication (e.g. cardiac pacemaker)

**Table 1:** Study objectives and outcome measures.

Study objective	Outcome measures
**Primary**	
To determine whether perfusion-based, ce-MRI can differentiate pleural malignancy from benign pleural disease with comparable or superior sensitivity and specificity to subjective CT or MRI morphology assessment	Diagnostic classification based on • MRI contrast enhancement pattern • CT morphology assessment • MRI morphology assessment
	Diagnostic assessment including pleural biopsy results

**Secondary**	
To determine whether there is a correlation between contrast enhancement pattern at MRI and tumour vascularity	• Mean Signal Intensity Gradient at ce-MRI • Tumour MVD based on Factor VIII immunostaining in FFPE pleural biopsies • Inter-observer agreement (Cohen’s Kappa) • Intra-observer agreement for ECE only (Cohen’s Kappa)
To determine the reproducibility of ECE, CT and MRI morphology	

**Exploratory**	
To determine whether there is an association between: 1. ce-MRI parameters and Survival 2. Tumour vascularity and Survival	• MSIG at ce-MRI • Overall Survival (months) • Tumour MVD (Factor VIII immunostaining in FFPE pleural biopsies) • Overall Survival (months)

ce-MRI; Contrast-enhanced Magnetic Resonance Imaging, CT; Computed Tomography, ECE; Early Contrast Enhancement, FFPE; Formalin-Fixed Paraffin-Embedded; MVD; Micro-vessel Density.

All patients underwent routine clinical work-up, [23] including contrast-enhanced CT and pleural biopsy by local anaesthetic thoracoscopy (LAT), where technically possible. Video-assisted thoracoscopic surgery (VATS) or image-guided pleural biopsy were permitted alternatives. Study procedures were limited to contrast-enhanced MRI prior to pleural biopsy and measurement of tissue MVD using surplus formalin-fixed, paraffin-embedded (FFPE) tissue, where available. The study protocol was approved by the West of Scotland Research Ethics Service (12/WS/0219, 13/WS/0240).

### 2.2 Sample size and assumptions

An *a priori* sample size calculation was not possible given the novel nature of the primary contrast-enhanced MRI outcome measure. A target sample size of 60 was deemed to be large enough for these methods to be developed and tested. Assuming a 50% incidence of MPM in the study cohort (based on our unit’s MPM incidence at LAT), 30 MPM patients would also allow a moderate correlation (r = 0.5) between the relevant secondary outcome measures to be detected with 80% power at a 5% two-sided level of statistical significance.

### 2.3 MRI acquisition

66 patients underwent 3-Telsa MRI (Siemens Magnetom Verio or Prisma^®^ (Erlangen, Germany)). Imaging protocols were developed in the first 6 patients, who did not receive contrast and are not included in any analyses. In the remaining 60 cases, isotropic, coronal T1-weighted, fat saturated, 3D spoiled gradient echo sequences (repetition time 2.8–3.2 ms, echo time 1–1.1 ms, field of view 400–440 mm, matrix 224 × 100, flip angle 9°, slice thickness 1.8–1.9 mm, no inter-slice gap) were acquired during a short breath-hold at end-inspiration. Images were acquired at baseline and at 40 s, 80 s, 4.5 min, 9 min and 13.5 min after injection of 0.1 mmol/kg gadobutrol contrast (Gadovist, Bayer, Germany) at 2 ml/s.

### 2.4 MRI analyses

#### 2.4.1 Morphology

Each case was classified as benign or malignant by two experienced thoracic radiologists (DS, GWC), based on morphology assessment alone. The presence or absence of established morphological features of PM, [24] including nodular or mediastinal pleural thickening, fissural nodularity, pleural thickening >1 cm and chest wall or diaphragmatic invasion, was used to classify patients. Both radiologists were blinded to the clinical and perfusion MRI data. A third thoracic radiologist (CN) provided a casting classification in discordant cases.

#### 2.4.2 Perfusion data

**Fig. 1:**
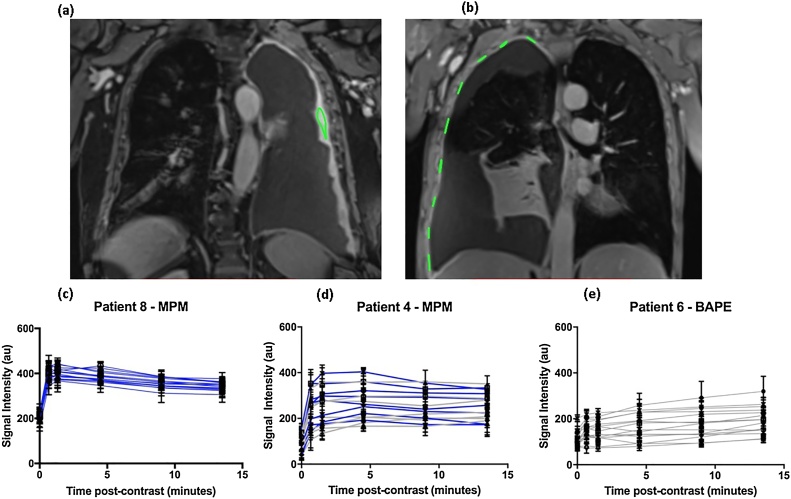
Contrast-enhanced, coronal, T1-weighted, fat-saturated, 3d spoiled gradient echo sequence Magnetic Resonance Images in two patients with Malignant Pleural Mesothelioma (MPM) with highlighted Regions of Interest (ROI). (**a**) demonstrates a patient with overtly nodular pleural disease. (**b**) demonstrates a patient with effusion-dominant, low volume pleural disease. Signal intensity/time curves for up to 15 ROI were plotted. (**c**) demonstrates an early peak in signal intensity at/before 4.5 min (Early Contrast Enhancement (ECE)) in all ROI in a patient with MPM. (**d**) demonstrates ECE in 9/15 ROI in a patient with MPM. (**e**) demonstrates no ECE in any ROI in a patient with Benign Asbestos Pleural Effusion (BAPE).

Perfusion analyses were performed by two senior respiratory physicians (ST and KGB), using OsiriX v5.8 (Pixmeo, Bernex, Switzerland). KGB’s results were used solely to assess inter-observer agreement. Repeat analyses by ST after a 2-month interval were used to assess intra-observer agreement. Images were anonymised and both operators were blinded to all other data. Up to 15 Regions of Interest (ROI) were defined at each time point (Fig. 1(a) and (b)), using a track-ball mouse and cursor, on representative areas of pleural disease. The pleura was defined as the visible structure running parallel with, and medial to the rib-cage and immediately contiguous with either aerated lung, pleural fluid or air (depending on the presence of a fully expanded lung, pleural effusion or pneumothorax, respectively at the imaged location). Care was taken to constrain the boundaries of the ROI to the parietal pleura, where possible, accepting that in cases where there was no pleural fluid or air separating parietal and visceral pleura this could not be guaranteed. Once the required number of ROI (minimum of 5 in patients with macro-nodular disease and 15 in patients with non-nodular disease) were defined on the 4.5-min post-contrast scan, these were electronically copied and pasted onto all other scans. Each scan was then visually assessed and each operator was asked to make minor adjustments to the position of each ROI to account for inconsistencies in the patient’s breath-hold and chest wall position, where required.

In patients with macro-nodular disease, a minimum of 5, and up to 15 ROIs were placed on pleural mass lesions (Fig. 1(a)). The number defined depended on the number of nodules; areas of necrosis were avoided. In the absence of macro-nodular disease, 15 ROIs were placed in all cases to ensure broad, random image sampling of the parietal pleura given the absence of visual indicators of pleural tumour. These ROI were defined consistently at anatomically similar locations in each patient (Fig. 1(b–d)), and were distributed across 3 coronal slices:

1. Midpoint slice: the slice with the largest continuous length of parietal pleura measured cranio-caudally. 9 ROIs were evenly distributed from cupula to costophrenic recess.
2. Anterior slice: the slice half-way from the midpoint slice to the most anterior slice where parietal pleura was identifiable. 3 ROIs were evenly distributed.
3. Posterior slice: the slice half-way from the midpoint slice to the most posterior slice where parietal pleura was identifiable. 3 ROIs were evenly distributed.

Signal intensity (SI) was measured within each ROI at each time point, generating ROI SI/time plots (Fig. 1). SI measurements were corrected for background signal noise using SI of extra-corporeal air. The time point that SI peaked in each ROI and that Mean SI peaked in each patient was recorded. This allowed computation of ROI SI gradient (ROISIG) for each ROI, and Mean SI gradient (MSIG) for each patient:

(meanorROI)SIgradient=(peakSI−baselineSI)÷timetopeakSI

MSIG was used to summarize ECE characteristics of each patient. ROISIG was used in a post-hoc analysis to define the characteristics of each ROI.

#### 2.4.3 Early contrast enhancement

To define a contrast enhancement pattern typical of malignancy, SI/time curves from the first 6 patients given gadobutrol with overtly malignant, nodular pleural disease were reviewed. An early peak in SI (occurring at or before 4.5 min) was identified in all 6 mean SI/time curves and 58/62 ROI SI/time curves from this cohort. This pattern was termed ECE (see Fig. 1(c) for an example). ROI SI/time curves in the remaining patients were then reviewed. ECE was deemed to be present, and the patient classified as Malignant, if at least one ROI SI/time curve demonstrated peak SI at/before 4.5 min (Fig. 1(d)). This criterion was chosen to replicate pleural biopsy interpretation where one malignant biopsy classifies a patient as malignant, even if all other biopsies are benign. If ECE was not identified in any ROI the patient was classified as benign (Fig. 1(e)).

#### 2.4.4 Post-hoc analyses regarding ROI signal intensity gradient (ROISIG)

Evidence of heterogeneous contrast enhancement (see Fig. 1(b)) prompted a post-hoc analysis to assess the contribution of benign (ECE-negative) ROI to the discriminant performance of ECE. We interpreted this as evidence of non-contiguous disease, commonly observed at thoracoscopy. Receiver Operator Characteristic (ROC) curves were plotted based on ROISIG for 1) all ROI in malignant cases relative to patients with benign disease and 2) only ECE-positive ROI in patients with malignancy relative to ROIs in benign cases.

#### 2.4.5 Combined MRI morphology and ECE

To determine the value of combined MRI morphology and ECE assessment, we classified patients using a 2-step approach: If morphological features of PM were present, the patient was classified as malignant, regardless of ECE findings. If MRI morphology was benign, then ECE results were examined. If ECE was present, then the patient was classified as malignant.

### 2.5 Contrast-enhanced CT

CT examinations were performed as part of routine clinical work-up, 55/60 had venous-phase CT and 5/60 had CTPA examinations. Scans were classified as malignant or benign based on the presence or absence, respectively, of established features of PM, [1] including nodular pleural thickening, pleural thickening >1 cm, mediastinal pleural thickening, enhancing pleural lesions, fissural nodularity, pleural mass or infiltration of mediastinal structures, chest wall or diaphragm, by the same experienced thoracic radiologists involved in MRI morphology assessment (DS, GWC +/− CN) using VuePACS v11.4 (Carestream Health Inc., NY) in blinded fashion.

### 2.6 Measurement of tissue microvessel density

Surplus FFPE pleural tissue biopsies in MPM patients only were examined by a Consultant Lung Pathologist (CD) to confirm that the tissue was representative of the patient’s histological diagnosis. Sections were cut on a digital microtome (4 μm thickness) and stained with Factor VIII immunostain (Leica Biosystems, UK, 1:200 dilution). Slides were digitized using Hamamatsu NDP (Hamamatsu, Welwyn Garden City). MVD was measured using quantitative image analysis software (Leica Biosystems, U.K.).

### 2.7 Statistical analyses

Data are described by mean (+/− SD) or median (inter-quartile range), depending on distribution. Contingency tables were used to calculate sensitivity, specificity, NPV and positive predictive value (PPV) of all imaging end-points. Diagnostic performance between methodologies was compared using McNemar’s test. Cohen’s Kappa statistic was used to assess inter- and intra-observer agreement. ROC curves were used to determine the diagnostic performance of ROISIG. MVD was correlated against MSIG using Spearman’s rho test. A p value ≤ 0.05 was considered statistically significant and values were adjusted for multiple comparisons using R v3.4. All other analyses were performed using Graphpad Prism v7 and SPSS v22.0.

## 3 Results

### 3.1 Recruitment

**Fig. 2:**
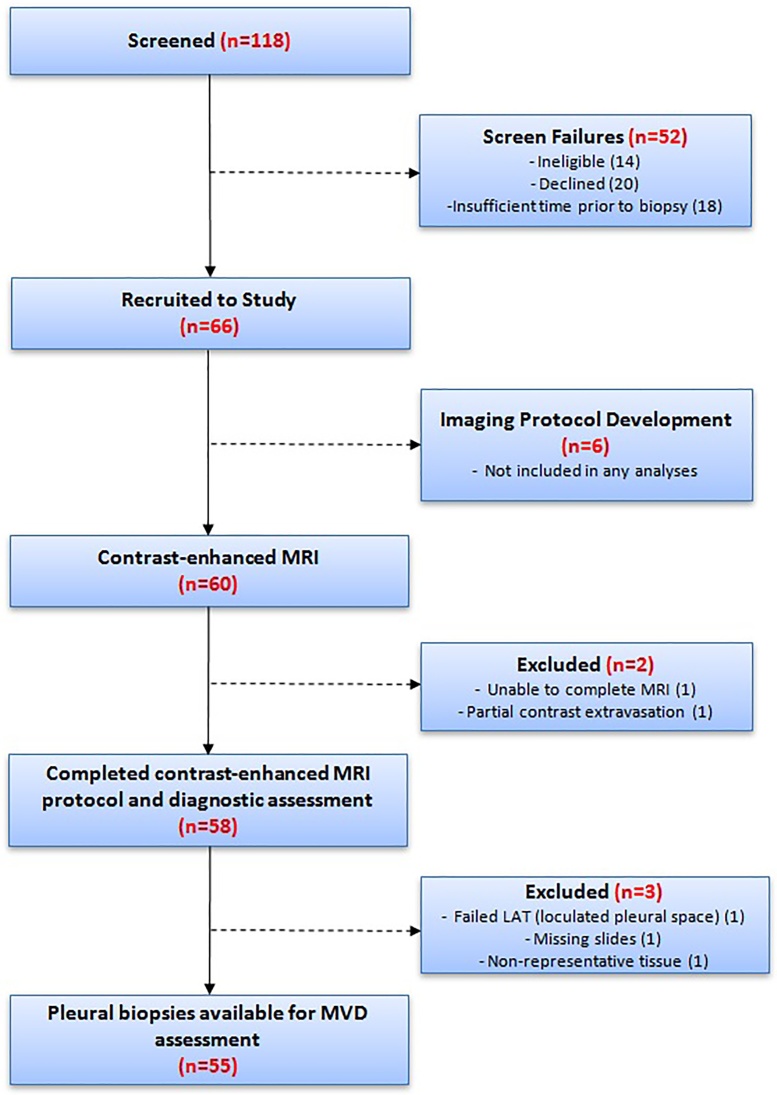
Study flow chart summarising screening, recruitment and protocol completion.

Recruitment is summarised in Fig. 2. 58/60 patients completed the planned contrast-enhanced-MRI protocol. 31/58 were diagnosed with MPM, all of whom had surplus tissue available for MVD measurement.

### 3.2 Demographics and clinical findings

Median patient age was 76 (70–81) years, 51/58 (88%) were male and 39/58 (67%) asbestos-exposed. 9/58 (16%) had a history of previous malignancy and 13/58 (22%) had pleural plaque disease.

36/58 (62%) had a final diagnosis of PM. 31/36 (86%) were diagnosed with MPM, of whom 65% (n = 20) had epithelioid MPM, 16% (n = 5) sarcomatoid MPM, 13% (n = 4) biphasic MPM and 6% (n = 2) had MPM not otherwise specified (NOS). 5/36 (14%) were diagnosed with secondary pleural malignancy, of whom 60% (n = 3) had metastatic breast cancer and 40% (n = 2) had metastatic lung cancer.

22/58 (38%) of patients had a final diagnosis of benign pleural disease. Benign pleural diagnoses included BAPE (50%, n = 11), tuberculous pleurisy (14%, n = 3), fibrothorax (9%, n = 2), rheumatoid pleurisy (9%, n = 2), reactive effusion associated with lung cancer (4.5%, n = 1), post-lobectomy effusion (4.5%, n = 1), secondary to pulmonary thromboembolism (4.5%, n = 1) and drug-related (4.5%, n = 1).

Final diagnoses were based on histology from LAT in 46/58 (79%), VATS in 7/58 (12%) and image-guided biopsy in 4/58 (7%). 1/58 (2%) were diagnosed based on radiology, MDT consensus and interval follow-up. All MPM cases were staged at regional MDT as I in 20/31 (64.5%), II in 0/31 (0%), III in 9/31 (29%) and IV in 2/31 (6.5%). Median overall survival for patients with PM was 20 months. Mean follow-up for patients with a benign pleural diagnosis was 20 (9) months.

### 3.3 Primary objective

MRI and CT were performed a median of 1 (1–7) days and 20 (13–34) days prior to pleural biopsy, respectively. At MRI, 49/58 (84%) patients had pleural thickening <10 mm (median pleural thickness 5 mm (4–7 mm)) and 47/58 (81%) lacked gross tumour nodules. Mean total scan duration was 35 (8) minutes. Contingency tables summarising CT, MR imaging and clinical results are presented in online Supplementary Table 1; associated diagnostic performance is summarised in Table 2.

**Table 2:** The diagnostic performance and reproducibility of CT morphology, MRI morphology and MRI-Early Contrast Enhancement (ECE) assessed in 58 patients with suspected Pleural Malignancy. Agreement was assessed using Cohen’s Kappa statistic. Diagnostic performance between groups is compared by McNemar’s test, adjusted for multiple comparisons. Statistically significant differences before and after adjustment are highlighted below.

	CT Morphology	MRI Morphology	MRI-ECE	Combined MRI Morphology-ECE
Sensitivity (95% CI)	56% (37–72%)	68% (48–83%)	83%* (61–94%)	92%** (67–100%)
Specificity (95% CI)	77% (60–89%)	85% (69–93%)	83% (68–91%)	78% (64–87%)
PPV (95% CI)	68% (47–84%)	77% (57–90%)	68% (47–84%)	55% (35–73%)
NPV (95% CI)	67% (50–80%)	78% (62–88%)	92% (78–97%)	97% (86–100%)
Inter-observer Agreement	0.65	0.593	0.784	N/R
Intra-observer Agreement	N/R	N/R	0.864	N/R

CT; Computed Tomography, MRI; Magnetic Resonance Imaging, CI; Confidence Interval, PPV; Positive Predictive Value, NPV; Negative Predictive Value, N/R; Not Recorded.

* Unadjusted p value .022 but adjusted p value .066 (MRI-ECE vs. CT morphology).

** Unadjusted p value .016 but adjusted p value .66 (Combined MRI morphology and ECE assessment vs. MRI morphology). For all other comparisons p > .05.

MRI perfusion analyses took a mean of 14 (3.5) minutes per patient; involving a mean of 14 (3) ROI per patient. 12/12 (100%) PM cases with macro-nodular disease were correctly classified as malignant based on ECE. 21/24 (87.5%) PM cases with non-nodular, effusion-dominant disease were correctly classified as malignant based on ECE. All three false negative cases based on ECE had MPM (see online Supplementary Table 2). Two cases were initially thought to be benign at LAT but developed progressive pleural thickening, prompting repeat biopsies and diagnoses of Epithelioid MPM (after 9 and 6 months respectively), with final classification as malignant. In both cases, this PM diagnosis was consistent with their initial positive ECE result. Both were classified as benign based on MRI morphology and one was classified as benign on CT morphology.

### 3.4 Secondary objectives

Results for inter- (and intra-) observer agreement regarding MR and CT end-points are summarised in Table 2. MSIG was significantly higher in patients with PM (0.58 (0.27–0.88) AU/s) than benign disease (0.2 (0.06–0.29) AU/s, p < .0001). In patients with MPM, there was a positive correlation between MSIG and MVD (r = 0.4258, p = .02, Fig. 4(b)). This relationship was preserved, and appeared more powerful when this correlation was confined to MPM patients without macro-nodular disease (r = 0.6594, p = .003, n = 18).

### 3.5 Exploratory objectives

#### 3.5.1 Micro-vessel density in FFPE tissue

Median tissue MVD in patients with MPM was 0.008761. Patients with a higher MVD (>0.008761) had significantly poorer median survival in comparison to those with lower MVD (10 months vs. 20 months, HR 2.723 (95% CI 1.093–6.784), p = .03), see (Fig. 4(c)).

#### 3.5.2 Mean signal intensity gradient at MRI

Median MSIG in patients with MPM was 0.533AU/s. Patients with a high MSIG (>0.533AU/s) had poorer median survival than those with a low MSIG (12 months vs. 20 months, HR 1.898 (95% CI 0.8349–4.316), p = 0.047 (Fig. 4(d)).

### 3.6 Post-hoc analyses regarding ROI signal intensity gradient

**Fig. 3:**
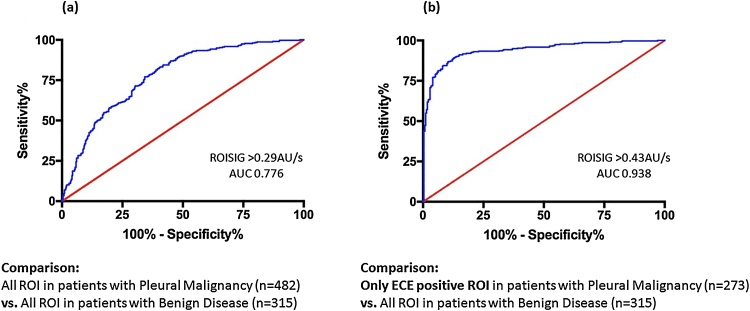
Receiver Operating Characteristic (ROC) curves of Region of Interest Signal Intensity Gradient (ROISIG) measured in 58 patients with suspected pleural malignancy (PM) using MRI. In (**a**), all ROI are included, including those who failed to demonstrate an Early Contrast Enhancement (ECE) pattern. In (**b**), only ROI demonstrating ECE in patients with PM are included, resulting in an improved discriminative performance.

A ROC curve plotted using only ROISIG data from ECE-positive ROI (n = 273) demonstrated optimum sensitivity (90% (95% CI 86–94%)) and specificity (86% (95% CI 81–89%)) at a threshold of ROISIG >0.43 AU/sec (AUC 0.938, (95% CI 0.918–0.957), p < .0001, see Fig. 3(a)). Using all ROISIG data (n = 482), regardless of ECE result, a ROISIG > 0.29 AU/sec provided optimum, but reduced sensitivity (71% (95% CI 67–75%)), specificity (68% (95% CI 63–73%)) and discriminatory performance (AUC 0.776 (95% CI 0.744–0.808), p < .0001, Fig. 3(b)).

**Fig. 4:**
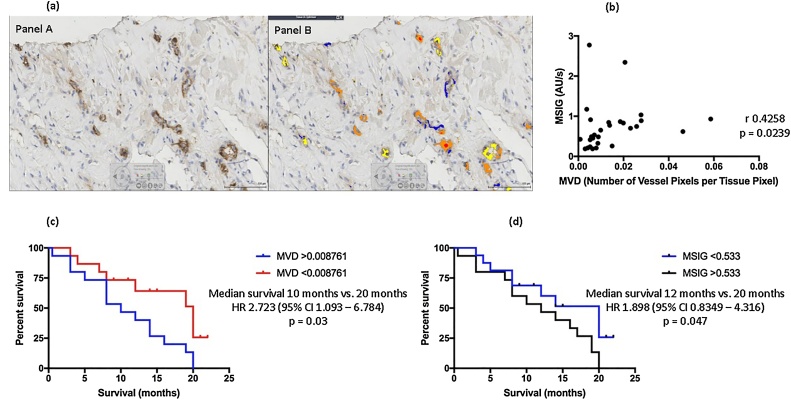
Paraffin-embedded pleural biopsies were stained with Factor VIII immunostain (**a**), Panel A). Computer software was used to detect the immunostain and calculate Microvessel Density (MVD) (**a**), Panel B). Mean Signal Intensity Gradient (MSIG) was measured by contrast enhanced-Magnetic Resonance Imaging in 31 patients with a final diagnosis of Malignant Pleural Mesothelioma (MPM) and correlated against MVD (**b**). Higher tumour MVD and MSIG in patients with MPM were associated with adverse survival (**c**) and (**d**), respectively).

## 4 Discussion

In this study, we have described the acquisition and preliminary diagnostic performance of a novel, semi-objective MRI biomarker of PM. ECE demonstrated higher diagnostic sensitivity (83%) and NPV (92%) than subjective CT morphology without perfusion analysis (56% and 67%, respectively) and MRI morphology (68% and 78%, respectively). After adjustment for multiple comparisons, these differences did not reach statistical significance. Combining MRI morphology with ECE resulted in slightly reduced specificity (78%) and PPV (55%) than with ECE alone (83% and 68%, respectively). ECE was associated with superior inter-observer agreement (κ 0.784) relative to CT (κ 0.65) and MRI morphology (κ 0.593).

ECE was defined as a peak in signal intensity at/before 4.5 min (270 s) post-gadolinium contrast. This time-point is later than the currently recommended timing for pleural CT acquisition following iodinated contrast administration (60 s). [[25], [26], [27], [28]] Tissue enhancement after gadolinium, which is an extracellular contrast agent, is dependent on several factors, including vascular perfusion, interstitial contrast uptake and clearance, and vascular permeability. [29,30] In this study, MSIG correlated positively with tissue MVD, suggesting this was a major contributor to the enhancement pattern observed. However, this analysis was confined to MPM cases and other factors, including delayed contrast clearance from peri-tumoural stroma may have contributed to the ‘delayed’ peak contrast enhancement observed. MPM is characterised by extensive peri-tumoural stroma, containing moderately cellular fibrous tissue. [31] Therefore these mechanisms should be further explored in future larger studies. Since ECE was defined based on observations in patients with macro-nodular tumour (MPM in 5/6, secondary PM in 1/6) it is possible that a different definition may perform better in patients with lower volume pleural tumour or in cancers of different types, in which different microvascular or other features predominate. However, the correlation we observed between tissue MVD and MSIG in MPM (r = 0.4258, p = 0.02, n = 28) appeared preserved, and indeed more powerful, when this analysis was confined to patients without macro-nodules (r = 0.6594, p = 0.003, n = 18). Furthermore, the 4.5 min SI peak observed here is concordant with a recent retrospective MRI study involving MPM patients, in which peak tumour enhancement occurred at 280 s post-gadolinium administration [32].

The diagnostic performance of ECE was similar to two earlier MRI morphology studies, in which pleural hyper-intensity on T1-weighted gadolinium-enhanced images accurately discriminated malignant from benign pleural disease. [33,34] These studies also reported similar false positive results in patients with tuberculous pleurisy, possibly reflecting angiogenesis in active tuberculous lesions. [35,36] However, it is important to note that the patients in these earlier studies had pleural thickening >10 mm, or pleural thickening that was significant, but not specifically measured. These older techniques therefore offered little additional diagnostic value over standard morphology, and have not entered clinical practice. DCE-MRI, mentioned earlier [16,18] is also poorly suited to early diagnosis, since it requires bulky disease for application. [20] Our data suggest that ECE is, potentially, a more useful diagnostic tool since the median pleural thickness in this study was 5 mm. Despite this, ECE was measurable in all patients and produced similar diagnostic performance to these earlier studies of more advanced pleural disease [33,34].

Similar to the findings in the present study, quantitative perfusion parameters at DCE-CT have been shown to correlate with tumour MVD. [[37], [38], [39]] Previous studies have reported on the potential utility of perfusion CT in the differentiation of benign from malignant pulmonary nodules. [40] In addition, results from a pilot study assessing DCE-CT in 13 patients with MPM, imaging a 5.5 cm axial extent of the thorax, reported correlation of contrast uptake with tumour burden and differential contrast uptake in patients undergoing chemotherapy versus observation alone. [7] However, the role of perfusion CT in the differentiation of PM from benign pleural disease has not been widely studied.

A potential advantage of perfusion CT over MRI- ECE, described here, is the greater availability of CT scanners and a quicker acquisition time. However, current perfusion CT methods do not allow complete assessment of the entire pleura, as is reported here using MRI, since perfusion CT is currently limited to defined sections of the thorax. Furthermore, high radiation exposure has so far limited clinical deployment of perfusion CT on a routine basis. [9,10]

PET-CT also offers potential advantages over the MRI methods reported here. In MPM, PET-CT out-performs both CT and MRI in assessment of nodal and distant metastases. [41] In addition, previous studies report elevated maximal standardised uptake values (SUVmax), in PM, relative to benign disease and high sensitivity and specificity (pooled sensitivity of 95% and specificity of 82%) in a meta-analysis [42], However, PET-CT specificity is reduced in TB pleuritis [43], as is reported here for MRI-ECE, and following talc pleurodesis [[44], [45], [46]]. PET-CT sensitivity is also reduced in early stage MPM, and epithelioid sub-types. [11,42] This significantly limits the utility of PET-CT as an early diagnostic test, particularly in effusion-dominant cases of MPM, which are most commonly of epithelioid origin.

The diagnostic performance of CT morphology reported here (sensitivity 56%, specificity 77%) is concordant with two recent studies (sensitivity 68%, specificity 78% [2] and sensitivity 68%, specificity 80%) [3] that retrospectively reported CT performance in routine practice, based on extracted reports. The current study involved prospective research-specific reporting by expert thoracic radiologists, but reported similar results. These are considerably inferior to those of older studies (e.g. sensitivity 92%, specificity 87%). [47] The same was true for our MRI morphology results (sensitivity 68%, specificity 85%) relative to previous studies, which reported sensitivity up to 96% and specificity up to 80%. [[47], [48], [49], [50]] These differences probably reflect earlier stage/lower volume PM in the current study, but highlight that morphology reporting is operator-dependent and subjective. Our finding of improved inter-observer agreement using ECE, a semi-objective measurement, is therefore an important outcome.

Similar to previous reports, increasing tumour MVD was associated with poorer survival in MPM. [51,52] Concordantly, increasing MSIG was also associated with worse outcomes. Our measurements were performed prior to any intervention that could have altered the pleural microcirculation, including pleurodesis or chemotherapy. This supports a conclusion that ECE is perfusion-based, although further studies focused on contrast enhancement mechanisms are required.

### 4.1 Potential clinical implications

While MRI is becoming increasingly available, the ability to assess patients with suspected MPM with thoracoscopy remains relatively limited to specialist centres. If validated in larger studies, ECE may improve the accuracy of pleural imaging in cases of suspected MPM. This may allow pathway rationalization, directing patients appropriately to specialist centres and early invasive sampling, including early thoracoscopy when MPM appears likely. The results of our ROISIG post-hoc analyses suggest that benign (ECE-negative) ROI in patients with PM negatively contribute to discriminant performance. This is consistent with a hypothesis that these data originate from areas of interspersed benign disease in patients with discontinuous malignant pleural lesions, which are commonly observed at thoracoscopy. Measurement of SI and assessment of ECE behavior across the entire pleura using a volumetric approach could be developed in future studies. Theoretically, this would allow the pleura to be treated as a ‘single ROI’. Pleural SIG, being a continuous variable like ROISIG, could then be applied at different thresholds depending on the clinical context. For example, a lower threshold (maximizing sensitivity) could be used in a pleural staging population vs. a higher threshold (maximizing specificity) for screening of asbestos-exposed persons for early MPM.

### 4.2 Study limitations

The principal limitation is the relatively small sample size, reflected in relatively wide confidence intervals around some measures of diagnostic performance. Nevertheless, we have assessed ECE in large number of individual ROI (n = 814) using a technique which appears reproducible. A second limitation is that although ECE is objectively defined, the user-defined ROI from which SI is measured are not. Our pre-defined strategy for ROI definition was devised to minimize variation and mirror thoracoscopic tissue sampling, but is a source of potential inconsistency. A volumetric approach could theoretically overcome this, but would require further development. An additional limitation is that we did not perform perfusion CT assessment for comparison with ECE, as all CT imaging in this study was performed as part of routine clinical work-up. However, as previously discussed, perfusion CT has not yet been widely studied in PM and is not routinely used in the assessment of patients with suspected PM in most centres at present. Finally, ECE analysis, although not laborious, is slower than morphology assessment. Technique evolution, including increased automation should reduce analysis time.

## 5 Conclusion

In this preliminary study, ECE appeared to be a sensitive, specific and reproducible biomarker of PM, with a high NPV, meriting further assessment in a large multi-centre study. ECE appears predominantly related to increased MVD in patients with MPM, although more precise information regarding contrast enhancement mechanisms is required. ECE methodology is applicable to patients with minimal pleural thickening, making this biomarker a potentially useful tool in effusion-dominant, low-volume PM, including early-stage MPM.

## References

[1] Metintas M., Ucgun I., Elbek O., Erginel S., Metintas S., Kolsuz M. (2002). Computed tomography features in malignant pleural mesothelioma and other commonly seen pleural diseases. Eur. J. Radiol..

[2] Hallifax R.J., Haris M., Corcoran J.P., Leyakathalikhan S., Brown E., Srikantharaja D. (2015). Role of CT in assessing pleural malignancy prior to thoracoscopy. Thorax.

[3] Tsim S., Stobo D.B., Alexander L., Kelly C., Blyth K.G. (2017). The diagnostic performance of routinely acquired and reported computed tomography imaging in patients presenting with suspected pleural malignancy. Lung Cancer.

[4] Miles K.A. (1999). Tumour angiogenesis and its relation to contrast enhancement on computed tomography: a review. Eur. J. Radiol..

[5] Yi C.A., Lee K.S., Kim E.A., Han J., Kim H., Kwon O.J. (2004). Solitary pulmonary nodules: dynamic enhanced multi-detector row CT study and comparison with vascular endothelial growth factor and microvessel density. Radiology.

[6] Ohno Y., Koyama H., Matsumoto K., Onishi Y., Takenaka D., Fujisawa Y. (2011). Differentiation of malignant and benign pulmonary nodules with quantitative first-pass 320-detector row perfusion CT versus FDG PET/CT. Radiology.

[7] Armato S.G., Labby Z.E., Coolen J., Klabatsa A., Feigen M., Persigehl T. (2013). Imaging in pleural mesothelioma: a review of the 11th International Conference of the International Mesothelioma Interest Group. Lung Cancer.

[8] García-Figueiras R., Goh V.J., Padhani A.R., Baleato-González S., Garrido M., León L. (2013). CT perfusion in oncologic imaging: a useful tool?. AJR Am. J. Roentgenol..

[9] Yamamuro M., Gerbaudo V.H., Gill R.R., Jacobson F.L., Sugarbaker D.J., Hatabu H. (2007). Morphologic and functional imaging of malignant pleural mesothelioma. Eur. J. Radiol..

[10] Gill R.R., Gerbaudo V.H., Sugarbaker D.J., Hatabu H. (2009). Current trends in radiologic management of malignant pleural mesothelioma. Semin. Thorac. Cardiovasc. Surg..

[11] Porcel J.M., Hernández P., Martínez-Alonso M., Bielsa S., Salud A. (2015). Accuracy of fluorodeoxyglucose-PET imaging for differentiating benign from malignant pleural effusions. Chest.

[12] Genestreti G., Moretti A., Piciucchi S., Giovannini N., Galassi R., Scarpi E. (2012). FDG PET/CT response evaluation in malignant pleural mesothelioma patients treated with talc pleurodesis and chemotherapy. J. Cancer.

[13] Bibby A.C., Tsim S., Kanellakis N., Ball H., Talbot D.C., Blyth K.G. (2016). Malignant pleural mesothelioma: an update on investigation, diagnosis and treatment. Eur. Respir. Rev..

[14] Treglia G., Sadeghi R., Annunziata S., Lococo F., Cafarotti S., Bertagna F. (2014). Diagnostic accuracy of 18F-FDG-PET and PET/CT in the differential diagnosis between malignant and benign pleural lesions. Acad. Radiol..

[15] Giesel F.L., Choyke P.L., Mehndiratta A., Zechmann C.M., von Tengg-Kobligk H., Kayser K. (2008). Pharmacokinetic analysis of malignant pleural mesothelioma-initial results of tumor microcirculation and its correlation to microvessel density (CD-34). Acad. Radiol..

[16] El Khouli R.H., Macura K.J., Kamel I.R., Jacobs M.A., Bluemke D.A. (2011). 3-T dynamic contrast-enhanced MRI of the breast: pharmacokinetic parameters versus conventional kinetic curve analysis. AJR. Am. J. Roentgenol..

[17] Mussurakis S., Buckley D.L., Drew P.J., Fox J.N., Carleton P.J., Turnbull L.W. (1997). Dynamic MR imaging of the breast combined with analysis of contrast agent kinetics in the differentiation of primary breast tumours. Clin. Radiol..

[18] Tan C.H., Paul Hobbs B., Wei W., Kundra V. (2015). Dynamic contrast-enhanced MRI for the detection of prostate cancer: meta-analysis. AJR. Am. J. Roentgenol..

[19] Fusco R., Sansone M., Filice S., Carone G., Amato D.M., Sansone C. (2016). Pattern recognition approaches for breast cancer DCE-MRI classification: a systematic review. J. Med. Biol. Eng..

[20] Giesel F.L., Bischoff H., von Tengg-Kobligk H., Weber M.-A., Zechmann C.M., Kauczor H.-U. (2006). Dynamic contrast-Enhanced MRI of malignant pleural mesothelioma: a feasibility study of noninvasive assessment, therapeutic follow-up, and possible predictor of improved outcome. Chest.

[21] Folkman J. (1971). Tumor angiogenesis: therapeutic implications. N. Engl. J. Med..

[22] Tsim S., Kelly C., Alexander L., McCormick C., Thomson F., Woodward R. (2016). Diagnostic and Prognostic Biomarkers in the Rational Assessment of Mesothelioma (DIAPHRAGM) study: protocol of a prospective, multicentre, observational study. BMJ Open.

[23] Hooper C., Lee Y.C.G., Maskell N., Pleural Guideline Group B.T.S. (2010). Investigation of a unilateral pleural effusion in adults: british thoracic society pleural disease guideline 2010. Thorax.

[24] Leung A.N., Müller N.L., Miller R.R. (1990). CT in differential diagnosis of diffuse pleural disease. AJR. Am. J. Roentgenol..

[25] Benamore R.E., O'Doherty M.J., Entwisle J.J. (2005). Use of imaging in the management of malignant pleural mesothelioma. Clin. Radiol..

[26] Raj V., Kirke R., Bankart M.J., Entwisle J.J. (2011). Multidetector CT imaging of pleura: comparison of two contrast infusion protocols. Br. J. Radiol..

[27] EVANS A.L., Gleeson F.V. (2004). Radiology in pleural disease: state of the art. Respirology.

[28] Helm E.J., Matin T.N., Gleeson F.V. (2010). Imaging of the pleura. J. Magn. Reson. Imaging.

[29] Padhani A.R., Dzik-Jurasz A. (2004). Perfusion MR imaging of extracranial tumor angiogenesis. Top. Magn. Reson. Imaging.

[30] García-Figueiras R., Padhani A.R., Beer A.J., Baleato-González S., Vilanova J.C., Luna A. (2015). Imaging of tumor angiogenesis for radiologists – part 1: biological and technical basis. Curr. Probl. Diagn. Radiol..

[31] O'Byrne K., Rusch V. (2006). Malignant Pleural Mesothelioma.

[32] Patel A.M., Berger I., Wileyto E.P., Khalid U., Torigian D.A., Nachiappan A.C. (2017). The value of delayed phase enhanced imaging in malignant pleural mesothelioma. J. Thorac. Dis..

[33] Falaschi F., Battolla L., Mascalchi M., Cioni R., Zampa V., Lencioni R. (1996). Usefulness of MR signal intensity in distinguishing benign from malignant pleural disease. AJR. Am. J. Roentgenol..

[34] Boraschi P., Neri S., Braccini G., Gigoni R., Leoncini B., Perri G. (1999). Magnetic resonance appearance of asbestos-related benign and malignant pleural diseases. Scand. J. Work. Environ. Health.

[35] Alatas F., Alatas O., Metintas M., Ozarslan A., Erginel S., Yildirim H. (2004). Vascular endothelial growth factor levels in active pulmonary tuberculosis. Chest.

[36] Matsuyama W., Hashiguchi T., Matsumuro K., Iwami F., Hirotsu Y., Kawabata M. (2000). Increased serum level of vascular endothelial growth factor in pulmonary tuberculosis. Am. J. Respir. Crit. Care Med..

[37] Kambadakone A., Yoon S.S., Kim T.-M., Karl D.L., Duda D.G., DeLaney T.F. (2015). CT perfusion as an imaging biomarker in monitoring response to neoadjuvant bevacizumab and radiation in soft-tissue sarcomas: comparison with tumor morphology, circulating and tumor biomarkers, and gene expression. AJR. Am. J. Roentgenol..

[38] Qin H.-Y., Sun H., Wang X., Bai R., Li Y., Zhao J. (2013). Correlation between CT perfusion parameters and microvessel density and vascular endothelial growth factor in adrenal tumors. PLoS One.

[39] Osimani M., Bellini D., Di Cristofano C., Palleschi G., Petrozza V., Carbone A. (2012). Perfusion MDCT of prostate cancer: correlation of perfusion CT parameters and immunohistochemical markers of angiogenesis. AJR. Am. J. Roentgenol..

[40] Ohno Y., Nishio M., Koyama H., Miura S., Yoshikawa T., Matsumoto S. (2014). Dynamic contrast-enhanced CT and MRI for pulmonary nodule assessment. AJR. Am. J. Roentgenol..

[41] Plathow C., Staab A., Schmaehl A., Aschoff P., Zuna I., Pfannenberg C. (2008). Computed tomography, positron emission tomography, positron emission tomography/computed tomography, and magnetic resonance imaging for staging of limited pleural mesothelioma. Invest. Radiol..

[42] Treglia G., Sadeghi R., Annunziata S., Lococo F., Cafarotti S., Bertagna F. (2014). Diagnostic accuracy of 18F-FDG-PET and PET/CT in the differential diagnosis between malignant and benign pleural lesions: a systematic review and meta-analysis. Acad. Radiol..

[43] Elboga U., Yılmaz M., Uyar M., Zeki Çelen Y., Bakır K., Dikensoy Ö. (2012). The role of FDG PET-CT in differential diagnosis of pleural pathologies. Revista Española De Medicina Nuclear E Imagen Molecular (English Edition).

[44] Genofre E.H., Vargas F.S., Acencio M.M.P., Antonangelo L., Teixeira L.R., Marchi E. (2009). Talc pleurodesis: evidence of systemic Inflammatory response to small size talc particles. Respir. Med..

[45] Vandemoortele T., Laroumagne S., Roca E., Bylicki O., Dales J.-P., Dutau H. (2014). Positive FDG-PET/CT of the pleura twenty years after talc pleurodesis: three cases of benign talcoma. Respiration.

[46] Nguyen N.C., Tran I., Hueser C.N., Oliver D., Farghaly H.R., Osman M.M. (2009). F-18 FDG PET/CT characterization of talc pleurodesis-induced pleural changes over time: a retrospective study. Clin. Nucl. Med..

[47] Hierholzer J., Luo L., Bittner R.C., Stroszczynski C., Schröder R.-J., Schoenfeld N. (2000). MRI and CT in the differential diagnosis of pleural disease. Chest.

[48] Knuuttila A., Kivisaari L., Kivisaari A., Palomäki M., Tervahartiala P., Mattson K. (2001). Evaluation of pleural disease using MR and CT. Acta Radiol..

[49] Falaschi F., Battolla L., Zampa V., Cioni R., Cambi L., Antonelli A. (1996). Comparison of computerized tomography and magnetic resonance in the assessment of benign and malignant pleural diseases. Radiol. Med..

[50] Coolen J., Vansteenkiste J., De Keyzer F., Decaluwé H., De Wever W., Deroose C. (2014). Characterisation of solitary pulmonary lesions combining visual perfusion and quantitative diffusion MR imaging. Eur. Radiol..

[51] Edwards J.G., Cox G., Andi A., Jones J.L., Walker R.A., Waller D.A. (2001). Angiogenesis is an independent prognostic factor in malignant mesothelioma. Br. J. Cancer..

[52] Kumar-Singh S., Vermeulen P.B., Weyler J., Segers K., Weyn B., Van Daele A. (1997). Evaluation of tumour angiogenesis as a prognostic marker in malignant mesothelioma. J. Pathol..

