# Supplementary material for: Early Contrast Enhancement: A novel magnetic resonance imaging biomarker of pleural malignancy
Source: Lung Cancer. 2018 Apr;118:48–56. doi: 10.1016/j.lungcan.2018.01.014 (PMC5884311; doi:10.1016/j.lungcan.2018.01.014)
Supplement: Supplementary file 1 [file mmc1.docx]

Supplementary Table 1. 2x2 Contingency tables for 58 patients with suspected Pleural Malignancy who underwent contrast-enhanced Computed Tomography (CT) morphological assessment, contrast-enhanced Magnetic Resonance Imaging (MRI) morphology assessment, MRI Early Contrast Enhancement (ECE) assessment and combined MRI morphology and ECE assessment followed by pleural biopsy.

|  | | **Final Pleural Diagnosis** | | |
| --- | --- | --- | --- | --- |
|  | | Malignant | | Benign |
| **CT morphology** | Malignant | 24 | | 7 |
|  | Benign | 12 | | 15 |
|  |  |  | |  |
|  | | **Final Pleural Diagnosis** | | |
|  | | Malignant | | Benign |
| **MRI morphology** | Malignant | 28 | | 5 |
|  | Benign | 8 | | 17 |
|  |  |  | |  |
|  |  | **Final Pleural Diagnosis** | | |
|  |  | Malignant | | Benign |
| **MRI ECE** | Malignant | 33 | | 7 |
|  | Benign | 3 | | 15 |
|  |  |  | |  |
|  |  | **Final Pleural Diagnosis** | | |
|  |  | Malignant | Benign | |
| **Combined MRI morphology and ECE assessment** | Malignant | 35 | 10 | |
|  | Benign | 1 | 12 | |

CT; Computed Tomography, MRI; Magnetic Resonance Imaging, ECE; Early Contrast Enhancement

Supplementary Table 2(a). 58 patients with suspected Pleural Malignancy (PM) underwent MRI for ECE assessment, CT scanning and pleural biopsy. 36/58 were diagnosed with PM. False negatives (*) case are highlighted.

Supplementary Table 2(b). 58 patients with suspected Pleural Malignancy (PM) underwent MRI for ECE assessment, CT scanning and pleural biopsy. 22/58 were diagnosed with benign disease. False positive cases (†) are highlighted.
